# Lung Expression of Macrophage Markers CD68 and CD163, Angiotensin Converting Enzyme 2 (ACE2), and Caspase-3 in COVID-19

**DOI:** 10.3390/medicina59040714

**Published:** 2023-04-06

**Authors:** Denis S. Ziablitsev, Marko Kozyk, Kateryna Strubchevska, Olena O. Dyadyk, Sergiy V. Ziablitsev

**Affiliations:** 1 Department of Pathophysiology, Bogomolets National Medical University, 01601 Kyiv, Ukraine; 2 Department of Internal Medicine, Corewell Health William Beaumont University Hospital, Royal Oak, MI 48073, USA; 3 Department of Pathologic and Topographic Anatomy, Shupyk National Healthcare University of Ukraine, 04112 Kyiv, Ukraine

**Keywords:** acute lung injury, exudative hemorrhagic pneumonia, fibrosis, CD68, CD163, ACE2, caspase-3, immunohistochemistry

## Abstract

*Background and Objectives:* The coronavirus (SARS-CoV-2) damages all systems and organs. Yet, to a greater extent, the lungs are particularly involved, due to the formation of diffuse exudative inflammation in the form of acute respiratory distress syndrome (ARDS) with next progression to pulmonary fibrosis. SARS-associated lung damage is accompanied by the pronounced activation of mononuclear cells, damage of the alveoli and microvessels, and the development of organized pneumonia. To study the expression of macrophage markers (CD68 and CD163), angiotensin-converting enzyme-2 (ACE2), and caspase-3 on the results of two fatal clinical observations of COVID-19. *Materials and Methods:* In both clinical cases, the female patients died from complications of confirmed COVID-19. Conventional morphological and immunohistochemical methods were used. *Results:* There was an acute exudative hemorrhagic pneumonia with the formation of hyaline membranes, focal organization of fibrin, stromal sclerosis, stasis, and thrombus formation in the lung vessels. Signs such as the formation of hyaline membranes, organization, and fibrosis were more pronounced in severe disease activity. The activation of CD68+/CD163+ macrophages could cause cell damage at an early stage of pneumonia development, and subsequently cause fibrotic changes in lung tissue. ACE2 expression in lung tissue was not detected in severe pneumonia, while in moderate pneumonia, weak expression was noted in individual cells of the alveolar epithelium and vascular endothelium. *Conclusions:* This finding could show the dependence of ACE2 expression on the severity of the inflammatory process in the lungs. The expression of caspase-3 was more pronounced in severe pneumonia.

## 1. Introduction

The severe acute respiratory syndrome caused by the Severe Acute Respiratory Syndrome Coronavirus 2 (SARS-CoV-2), also known as the coronavirus infectious disease (COVID-19), has affected every country [1]. As of 4 January 2023, there were more than 655.6 million cases of COVID-19 and more than 6.6 million deaths worldwide [2].

SARS-CoV-2 damages almost all systems and organs, but the lungs suffer the greatest damage [3]. At the first stage of coronavirus lung damage, diffuse exudative inflammation in the form of acute respiratory distress syndrome (ARDS) is described [4]. Next, the exudative stage progresses to a proliferative stage with pulmonary fibrosis. SARS-associated lung damage is typically accompanied by the appearance of many mononuclear cells in the interstitium, intra-alveolar hemorrhages, and purulent inflammation, leading to the development of organized pneumonia [5].

The incidence of ARDS in patients with COVID-19 is 29%, and the mortality rate is up to 15% [3]. The basis of the development of ARDS is an acute disturbance of pulmonary perfusion with an increase in vascular permeability [6] and a hyperergic response of the immune system with a “cytokine storm” [7]. The exact mechanisms of lung damage in COVID-19 are not yet fully understood [8].

The widely accepted hypothesis of the dysregulation of mononuclear cells might explain the hyperinflammation associated with COVID-19 [8,9]. The hyperactivation of monocytes, which is observed in patients with COVID-19, is facilitated by the delayed production of interferon I, which activates the release of monocyte chemo-attractants by the alveolar epithelium and activates the recruitment of blood monocytes in the lungs [8]. Next, monocytes differentiate into pro-inflammatory macrophages through the activation of Janus kinase (JAK) and signal transducer and activator of transcription (STAT) pathways, Toll-like receptor 4 (TLR4)–TRAF6–NF-κB, TLR7 pathways, or NLRP3 inflammasome activation, etc. [8].

Even after the first epidemic of the coronavirus infectious disease (COVID) in 2003, a functional receptor, which is necessary for the penetration of the coronavirus into host cells, was identified [10]. Angiotensin-converting enzyme-2 (ACE2) consists of 805 amino acids and is a transmembrane glycoprotein; its role is to convert angiotensin 1 into inactive angiotensin 1–9, as well as angiotensin 2 into angiotensin 1–7 [11,12]. The latter counteracts such effects of angiotensin II as vasoconstriction, proliferation, fibrosis, and inflammation [13]. In the lungs, during inflammation, angiotensin 1–7 suppresses the infiltration of inflammatory cells, the release of proinflammatory cytokines, improves oxygenation, reduces apoptosis of the alveolar epithelium, and suppresses the proliferation and migration of fibroblasts [13,14]. ACE2, a direct target for the coronavirus, is an important component of the tissue renin–angiotensin system and has a number of protective effects during acute inflammation [15]. ACE2 is expressed by macrophages of the subcapsular and splenic marginal zone of lymph nodes [16]. ACE2 expression by macrophages can be triggered by pro-inflammatory signals such as interferon I [17].

An important role in the development of pneumonia in COVID-19 belongs to apoptosis, which has both adaptive and damaging effects [18]. Thus, apoptosis of pulmonary polymorphonuclear leukocytes suppresses the inflammation and the development of ARDS, while Fas/FasL-mediated apoptosis of alveolar epithelial cells contributes to acute lung injury and pulmonary fibrosis [19]. Induced pulmonary inflammation in wild-type mice was accompanied by significant activation of caspase-3, which is a key enzyme in the initiation of the apoptotic signal [20].

In this study, the expression of macrophage markers (CD68 and CD163), ACE2, and caspase-3 was investigated based on the results of two fatal clinical observations of COVID-19.

## 2. Materials and Methods

### 2.1. Clinical Case 1

Case 1 was a 47-year-old female patient who was 33 weeks pregnant. She had a past medical history of chronic hypertension with superimposed preeclampsia and maternal medical care associated with intrauterine fetal hypoxia. The patient presented with a dry cough and a temperature of 36.6 °C. On admission, an increase in productive cough and shortness of breath were noted. The SARS-CoV-2 coronavirus was detected in the blood by the polymerase chain reaction method; the content of antibodies in the blood was not determined. The patient was unvaccinated. Her oxygen saturation on admission was 87%. One day after admission, the patient had conservative childbirth, which lasted 5 h and 45 min. Immediately after delivery, her temperature decreased to 35.0 °C and oxygen saturation dropped to 65%. The patient was intubated; however, she developed sudden cardiac arrest, after which she passed despite resuscitation efforts.

The autopsy revealed pleural cavities on the left (0.3 L) and on the right (0.4 L) containing bloody fluid. The mucous membrane of the larynx, trachea, and bronchi was gray-pink, smooth, and shiny with small focal-point hemorrhages. The lungs were crimson-red in color and dense in texture.

### 2.2. Clinical Case 2

Case 2 was a 67-year-old female patient with a past medical history of heart failure with reduced ejection fraction, ischemic heart disease, and breast cancer status post-mastectomy with radiation, who was admitted with a diagnosis of acute respiratory viral infection. On admission, the patient had a heart rate of 80 beats per minute, a blood pressure of 120/80 mm Hg, and a temperature of 36.6 °C. Objectively, the general condition of the patient was satisfactory, and the skin and mucous membranes were pale pink in color. Cardiac dullness was within normal limits and heart sounds were weakened. Course crackles were noted bilaterally. Her abdomen was soft and not painful to palpation; the liver was on the edge of the costal arch, and the spleen was not enlarged. The SARS-CoV-2 coronavirus was detected in the blood by the polymerase chain reaction method; the content of antibodies in the blood was not determined. The patient was unvaccinated.

Two days after admission, the patient’s condition had worsened; she complained of weakness, pain behind the sternum and acrocyanosis. Her breathing was hard, wet rales were noted on both sides, and her blood pressure was 60/40 mm Hg. The patient rapidly deteriorated and had cardiopulmonary arrest. Despite resuscitation efforts, clinical death was documented.

On autopsy, the right lung weighed 615 g and the left 375 g. The surface of the lungs was gray-pink with the presence of multiple areas of a dark purple color. On the section of the lung parenchyma, gray-red segments alternated with dark purple ones. Above the surface of the incision, the thickened walls of the bronchi protruded, and a significant amount of dark-red viscous content was released from the lumen. The mucous membrane of the bronchial tree throughout was gray-red with the presence of gray-red viscous contents in the lumen.

### 2.3. Morphological Studies

Pieces of lung tissue from different areas were fixed in a 10% solution of neutral buffered formalin (pH 7.4) for 24–36 h. After fixation in formalin, tissue pieces were embedded in paraffin. Serial histological sections with a thickness of 2–3 μm were made from paraffin blocks on a rotary microtome NM 325 (Thermo Shandon, Knutsford, Cheshire, UK). Serial paraffin sections were stained with hematoxylin and eosin. Immunohistochemical examination (IHC) was performed using monoclonal antibodies against ACE2 (anti-ACE2; clone 4G5.1; Sigma-Aldrich MABN59; replaces MAB5676) manufactured by EMD Millipore Corporation, Temecula, CA, USA; monoclonal anti-bodies against caspase-3 (Caspase 3 Monoclonal Antibody, clone74T2; ThermoFisher Scientific, City, USA); and monoclonal antibodies against CD68 (Clone KP-1, Master Diagnostica, City, Spain) and CD163 (Anti-CD163 antibody [OTI2G12] (ab156769), Abcam, City, USA). IHC was performed on Super Frost Plus (Menzel, Berlin, Germany) adhesive slides. For the high-temperature treatment of antigens’ epitopes, citrate buffer (pH 6.0) and EDTA buffer (pH 8.0) were used. The Master Polymer Plus Detection (Peroxidase, DAB chromogen) detection system (Master Diagnostica, Granada, Spain) was utilized. Sections were additionally stained with hematoxylin. Microscopic analysis and photo archiving were carried out using light-optical microscopes “ZEISS” (Köln, Germany) with the processing system “Axio Imager. A2” “ZEISS” (Köln, Germany) with 5-, 10-, 20-, and 40-times objective magnification; 1.5-times binocular attachment; and 10-times eyepiece with ERc 5s cameras. “Carl Zeiss” Primo Star and Axiocam 105 color and light optical microscope “Olimpus BX 40” was additionally equipped with an “Olimpus C3030-ADU” digital camera and “Olimpus DP-Soft” software (Olimpus, Feasterville, PA, USA).

## 3. Results

### 3.1. Clinical Case 1

**Figure 1:**
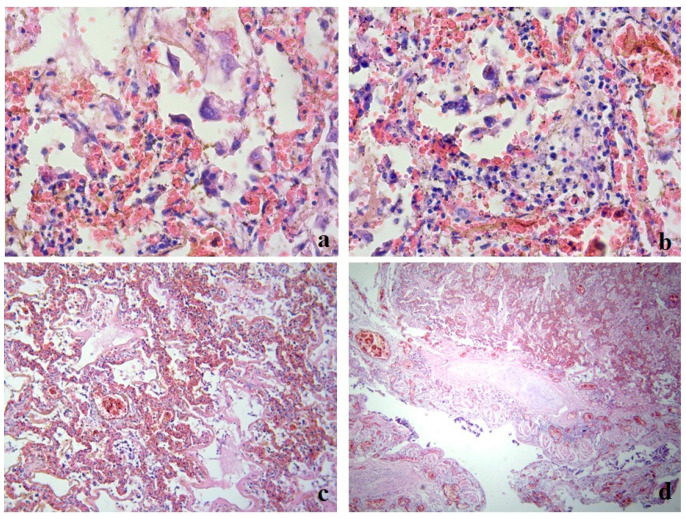
Clinical case 1. Lung tissue; staining with hematoxylin and eosin. (**a**) Hemorrhages, desquamated epithelium in the alveolar space, formation of multinucleated cells; ×400. (**b**) Alveolar macrophages in the alveolar space, lympho-monocitrate infiltrate; ×400. (**c**) Parenchyma, numerous hemorrhages, formation of hyaline membranes, stasis, thrombosis of small vessels; ×100. (**d**) Bronchus wall, exfoliated epithelium, focal layering of fibrin on the surface, serous-hemorrhagic pneumonia, vascular thrombosis; ×100.

During the pathomorphological examination of the respiratory system, it was established that the patient had virus-induced changes in the epithelial cells of the trachea, bronchi, and alveoli. The epithelial cells had signs of luminal transformation and an increase in the size of nuclei and cytoplasm. In the lung tissue, diffuse pleurisy, alveolar damage, and areas of desquamation with the formation of multinucleated cells in the space of the alveoli were noted (Figure 1a).

Clusters of giant macrophage cells were noted in the alveoli and focally in the stroma, which indicated the presence of an active response of innate cellular immunity (Figure 1b).

Signs of acute exudative inflammation in the form of fibrin seepage and the formation of hyaline membranes, focal zones of fibrin organization, areas of squamous metaplasia, and initial sclerosis of the stroma were noted. The microcirculatory bed had stasis and thrombus formation (Figure 1c).

The changes in the stromal component were observed in the form of diffuse damage to the alveolar chain, local cellular immune response, and pronounced changes in the vessels of the microcirculatory bed.

In the trachea and bronchi, there was marked blood, large areas of desquamated epithelium, fibrinous–necrotic inflammation, layering of fibrin in the affected areas, fibrin on the surface, blood in the vessels of the submucosal layer, and thrombus formation in the vessels (Figure 1d).

**Figure 2:**
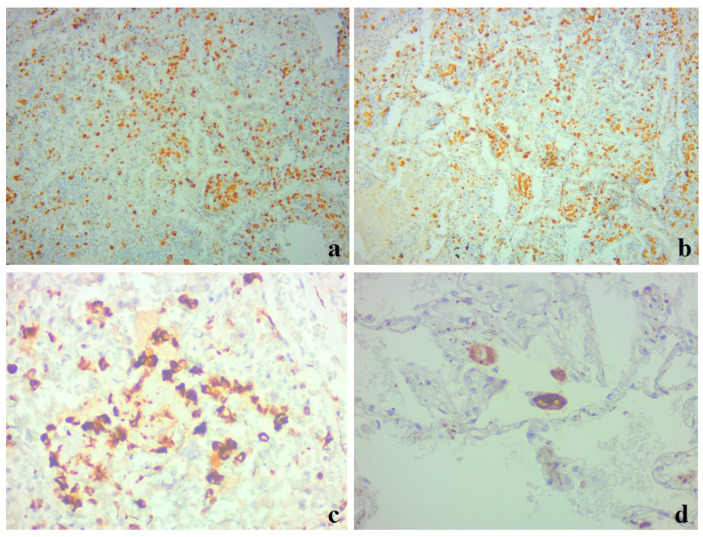
Clinical case 1. Lung tissue. Immunohistochemistry for CD68 (**a**,**c**,**d**) and CD163 (**b**). (**a**,**b**) Large number of immunopositive macrophages in areas of cellular infiltrates; ×100. (**c**) Large CD68+ macrophages in the alveolar space; ×400. (**d**) Giant single CD68+ macrophages in the alveolar space; ×400.

A large number of immunopositive CD68+ macrophages were segmentally located in the areas of cell infiltration (Figure 2a). The majority of these cells also had immunopositive staining for the hemoglobin–haptoglobin complex receptor CD163 (Figure 2b).

Clusters of large CD68+ cells represented macrophages (Figure 2c) and were detected in the alveolar space (Figure 2d). A large number of small CD68+ cells were also detected in the interalveolar membranes (see Figure 2c,d).

### 3.2. Clinical Case 2

**Figure 3:**
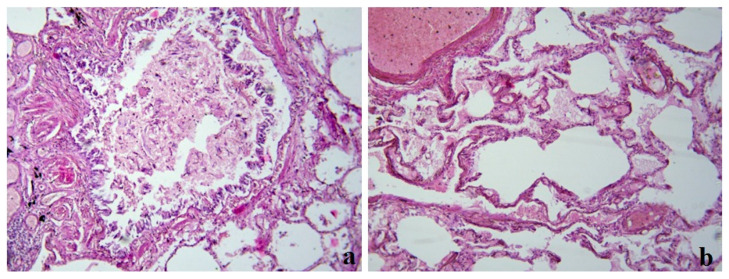
Clinical case 2. Lung tissue; staining with hematoxylin and eosin; ×100. (**a**) Bronchus wall; desquamation of the epithelium, fibrin and exfoliated cells in the lumen, peribronchial fibrosis. (**b**) Edema in the lung tissue, luminal transformation of epithelial cells, and thrombus formation.

During the pathomorphological examination of the respiratory system, viral-induced changes in the epithelial cells of the trachea, bronchi, and alveoli were noted. In the cells of the bronchial epithelium, desquamation of the epithelium and exfoliated cells were observed (Figure 3a). In the lung tissue, there was unevenly expressed pleurisy, areas of varying degrees of alveolar damage, focal desquamation of epithelial cells, and peribronchial fibrosis (Figure 3b).

**Figure 4:**
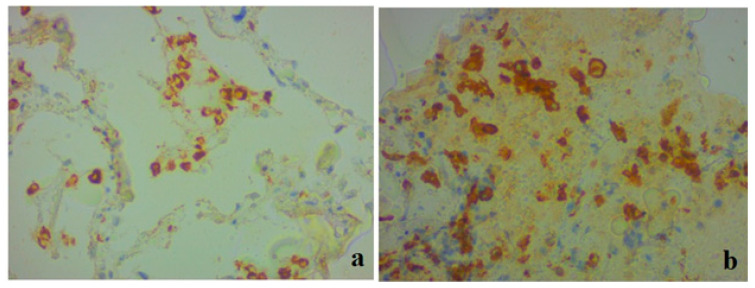
Clinical case 2. Lung tissue. Representative results of immunohistochemistry for CD68 (**a**) and CD163 (**b**); ×400. (**a**) Positive CD68+ macrophages in alveolar space, interstitium, and interalveolar membranes. (**b**) Accumulation of positive CD163+ macrophages in the alveolar space.

Immunopositive CD68+ and CD163+ macrophages of various sizes were observed in the alveolar space, interstitium, and interalveolar membranes (Figure 4).

**Figure 5:**
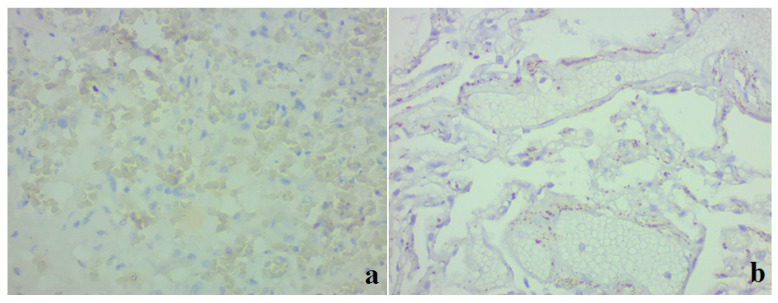
Lung tissue. Representative results of an immunohistochemical study on ACE2; ×400. (**a**) Clinical case 1; lack of immunospecific staining in lung tissue. (**b**) Clinical case 2; positive staining of ACE2 in individual areas in the interalveolar membranes, in a part of endothelial cells, focally in individual alveolocytes.

We also conducted a study using a monoclonal antibody against ACE2. In clinical case 1, a complete absence of immunopositive staining was noted (Figure 5a). In case 2, weak to moderately pronounced immunopositive staining of ACE2 was noted along the cells of the alveolar epithelium, as well as in separate areas in the walls of the interalveolar membranes (Figure 5b). A positive expression was also noted in endothelial cells, which had signs of stasis and thrombus formation.

**Figure 6:**
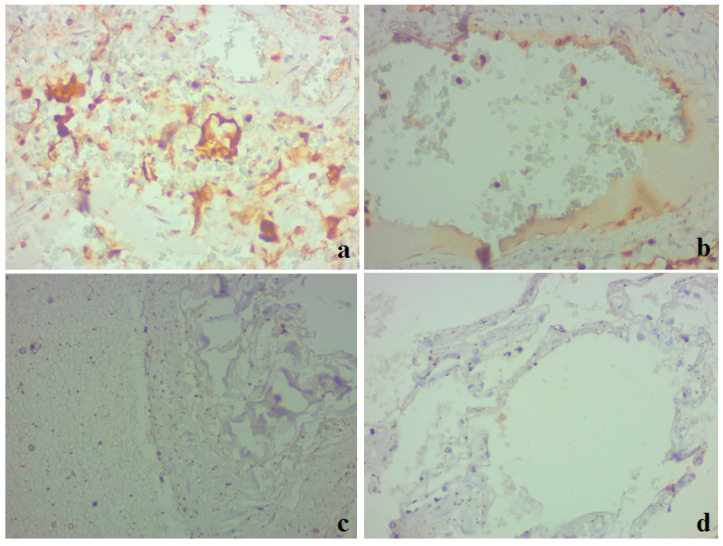
Lung tissue. Results of an immunohistochemical study utilizing caspase-3; ×400. (**a**,**b**) Clinical case 1; pronounced positive staining in alveolocytes, bronchial epithelium, macrophages, and individual endothelial cells (**b**). (**c**,**d**) Clinical case 2; lack of immunospecific staining in lung tissue.

IHC with the marker of apoptosis protein caspase-3 was noted in the epithelial cells of the bronchial epithelium, alveolocytes, macrophages (Figure 6a), and vascular endothelial cells (Figure 6b) in clinical case 1. No IHC staining with caspase-3 was detected in clinical case 2 (Figure 6c,d).

## 4. Discussion

Thus, morphological studies of the lungs in patients with confirmed COVID-19 showed the presence of acute exudative hemorrhagic pneumonia with the formation of hyaline membranes in the alveoli, the focal organization of fibrin, stromal sclerosis, stasis, and thrombus formation in vessels. At the same time, the formation of hyaline membranes and fibrosis were more pronounced in case 1, which corresponds to a severe course of the disease.

In both cases, the activation of CD68+/CD163+ macrophages took place. Giant alveolar macrophages accumulated in the alveolar space. At the same time, the expression of ACE2 in the lung tissue in clinical case 1 was not detected, while in case 2, a weak expression was noted in individual cells of the alveolar epithelium and vascular endothelium. The expression of caspase-3 was noted in case 1 in the bronchial epithelium, cellular infiltrates, and vascular endothelium. These results coincided with the data of an Italian study, according to which all patients who died from COVID-19 had diffuse alveolar damage with pneumocyte necrosis, hyaline membranes, pronounced interstitial and intra-alveolar edema, platelet–fibrin thrombi, and a pronounced inflammatory infiltrate in the lumen of the alveoli with a large number of macrophages [21]. The alveolar damage in COVID-19 pneumonia, in comparison with other etiological factors, might be explained by pseudo-palisaded histiocytic hyperplasia of CD68+ and other immune cells (CD4, CD8, CD20, AE1/AE3), and active microthrombus formation in the lungs [22].

Early stages of COVID-19 pneumonia usually include cellular hyperplasia of type II pneumocytes, accompanied by strong nuclear expression of phosphorylated STAT3 [23]. Macrophages of the alveolar space showed a peculiar pro-inflammatory phenotype (CD68, CD11c, CD14, CD205, CD206, CD123/IL3AR, and PD-L1). At the same time, the formation of hyaline membranes and fibrosis of lung tissue was not detected. Therefore, it can be assumed that SARS-CoV-2 infection leads to the activation of macrophages in the early stages of inflammation.

Pathomorphological studies of the lungs of those patients who died from COVID-19 showed the presence of pneumocytes, CD68+ macrophages, and CD3+ T cells with the activation of STAT6 expression in the inflammatory infiltrates [24]. Patients who died from COVID-19 showed a significant increase in CD4, CD68, and CD138 [25].

An analysis of the cytopathological features of bronchoalveolar lavage from patients with COVID-19, compared to patients who had acute respiratory distress syndrome caused by other pathogens, showed a higher number of neutrophils and a lower percentage of macrophages and lymphocytes [26]. In contrast, the expression of CD68+ monocytic multinucleated giant cells was significantly increased in our patients. Therefore, it is these cells that accompany the development of hyperimmune inflammation in COVID-19. Important results were obtained in the study of CD163+ macrophages, which are considered to be profibrotic cells (phenotype M2) [27]. The CD163 receptor is widely known as a scavenger receptor of the hemoglobin–haptoglobin complex, which is abundantly expressed in macrophages [28].

CD163+ macrophages might be activated by hemolysis [29]. CD163+ macrophages are of monocyte origin and accumulate in pulmonary infiltrates in the ARDS of COVID-19, demonstrating a profibrotic M2 transcriptional phenotype. A gene set analysis showed a significant similarity of CD163+ macrophages associated with COVID-19 to profibrotic macrophage populations found in idiopathic pulmonary fibrosis [30]. There are data on the specific stimulation by SARS-CoV-2 of profibrotic changes in macrophages, which triggers severe fibroproliferative ARDS [31]. Based on these considerations, the activation of CD163+ macrophages detected in our studies may explain the active profibrotic changes, especially in clinical case 1. These findings can be supported by the results of the detection of aberrant CD163+ monocytes in the blood, respiratory tract, and alveoli of patients with a severe form of COVID-19 [32]. The clinical significance of the activation of CD163+ macrophages is demonstrated by increased levels of soluble CD163 (sCD163) in the blood plasma of patients with COVID-19 [33].

The macrophage reactions in severe COVID-19 are similar to “hyperferritinemic syndromes”, including macrophage activation syndrome (MAS), adult Still’s disease, catastrophic antiphospholipid syndrome, and septic shock [34].

Uncontrolled activation of macrophages, together with cytotoxic T-cells and natural killers (NK), forms the basis of hemophagocytic lymphohistiocytosis (HLG), which is also accompanied by cytokine hypersecretion and immune-mediated organ damage [35]. GLH can be primary and secondary, which develops in hemoblastosis, viral infections, and rheumatic diseases. Increased expression of CD163 and the plasma content of CD163 are considered sensitive markers of MAS [36].

ACE2 is a key cellular protein by which SARS-CoV-2 enters cells and reproduces in them [37]. In the lung parenchyma, ACE2 protein was detected on the apical surface of a small number of type II alveolar epithelial cells. This particular feature of ACE2 expression contributes to the penetration and multiplication of the virus [38]. The level of ACE2 expression in the lungs is positively correlated with an increased risk of severe infection and complications in COVID-19, as well as with an increase in macrophage infiltration and CD163 expression [39]. Therefore, the predominance of the profibrotic phenotype of macrophages may determine the severity of the disease course.

It is instructive that the product of ACE2 – angiotensin 1–7 is able to reduce the expression of pro-inflammatory cytokines TNF-α and IL-6 and increase the expression of anti-inflammatory cytokines IL-4 and IL-10, due to the effect on the polarization of macrophages from the M1 phenotype to the M2 phenotype [40]. This indicates a close connection between the proinflammatory imbalance of the angiotensin system and the formation of the profibrotic phenotype of macrophages.

In autopsy samples of patients with COVID-19 pneumonia, ACE2 was detected in rare ciliated epithelial and endothelial cells of the trachea, but not in the lungs [41]. The same results were obtained in our study—the expression of ACE in the severe form of pneumonia was absent, while in the case of pneumonia of moderate severity (case 2) the ACE-2 expression was weak and related mainly to single cells of the epithelium and endothelium. On the other hand, according to another study, the presence of ACE2 was demonstrated in alveolar type II cells, as well as in newly formed interstitial capillaries [42].

Such data discrepancies, in our opinion, can be explained by different stages of acute lung inflammation. In our previous experimental studies with modeling of acute bronchopulmonary inflammation in aspiration lipopolysaccharide-induced pneumonia, different levels of ACE2 expression were shown during the clinical course [43]. The exudative phase of acute inflammation was accompanied by an inhibition of ACE2 expression in bronchial epithelial cells, alveolocytes type II, and the vascular endothelium. During proliferation and fibrosis, the expression of ACE2 was increased [44].

Apoptosis activity, which was evaluated by the pulmonary expression of caspase-3, showed a significant activation in the severe course of the disease in our studies that coincides with known data [45]. It has also been shown that both the gene expression and the serum level of caspase-3 are positively correlated with the severity and pro-inflammatory markers of severe ARDS in COVID-19 [46]. In another study of ours, which analyzes experimental lipopolysaccharide-induced aspiration pneumonia, it was shown that the content of both forms of caspase-3 (pro-caspase-3 and active caspase-3) increased [47]. The active form of caspase-3 is an effector enzyme that sets the cell on the path of apoptosis [18,48,49].

Our studies show that, at the stage of acute inflammation, macrophages, fibroblasts, and bronchial epitheliocytes showed the highest caspase-3 activity, while at the stage of fibrosis, bronchial epitheliocytes had the highest expression [47]. Our clinical study revealed positive staining for caspase-3 in the severe form of COVID-19, which was noted in the epithelial cells of the bronchial epithelium and part of the alveolocytes, macrophages of the cellular infiltrates, as well as the vascular endothelium. Thus, apoptosis of pro-inflammatory macrophages of the M1 type at the early stage of inflammation precedes the replacement of their phenotype with M2 at the stage of fibrosis. The significant activation of caspase-3 in the vascular endothelium might be explained by the direct action of the S1 subunit of SARS-CoV2 [50]. On the contrary, the activation of vascular endothelium apoptosis in the experimental model allows us to consider it as a non-specific result of hyperimmune pulmonary inflammation [47].

In clinical case 2, we found no signs of apoptosis activation. The course of pneumonia and pulmonary fibrosis were less severe, while the high expression of ACE2 indicated the resolution of the acute inflammatory process, which likely caused the absence of caspase-3 expression. In a previous experimental study, there was a decrease in the activity of both pro-caspase 3 and caspase-3 during the decrease in the inflammatory process in the bronchial epithelium, endothelium, fibroblasts, and cells of interstitial follicles [47].

## 5. Conclusions

The performed postmortem examinations of the lungs showed the presence of acute exudative hemorrhagic pneumonia with the formation of hyaline membranes in the alveoli, the focal organization of fibrin, sclerosis of the stroma, stasis, and thrombus formation in the vessels. Hyaline membranes, organization, and fibrosis were more pronounced in the severe form of the disease. The pathologic causes of respiratory failure were exudate in the air spaces of the lungs, exfoliated epithelium, as well as fibroproliferative remodeling of the lung parenchyma. The activation of CD68+/CD163+ macrophages could cause cell damage at an early stage of pneumonia development, and subsequently cause fibrotic changes in lung tissue. ACE2 expression in lung tissue was not detected in severe pneumonia, while in moderate pneumonia, weak expression was noted in individual cells of the alveolar epithelium and vascular endothelium. This could reflect the dependence of ACE2 expression on the severity of the inflammatory process in the lungs. The expression of caspase-3 was more pronounced in severe pneumonia.

### Study Limitations

The description of two clinical cases is not sufficient to understand all the pathological processes in COVID-19-associated pneumonia. The set of markers utilized was limited; however, in our opinion, the conducted research gives a perspective on the understanding of macrophages and apoptotic process and justifies the feasibility of continuing scientific research in this direction.

## Data Availability

Not applicable.
